# Machine learning for the diagnosis of fibromyalgia based on magnetic resonance imaging

**DOI:** 10.1371/journal.pone.0340899

**Published:** 2026-02-02

**Authors:** Zhangying Zeng, Weihang Liao, Xuemei Wu, Xinyue Liao, Yating Ou, Lan Zhao, Li Zhao, Daoshu Luo, Feng Wang

**Affiliations:** Laboratory of Clinical Applied Anatomy, the School of Basic Medical Sciences, Fujian Medical University, Key Laboratory of Brain Aging and Neurodegenerative Diseases of Fujian Province, Fuzhou, China; Chengdu University of Traditional Chinese Medicine Wenjiang Campus: Chengdu University of Traditional Chinese Medicine, CHINA

## Abstract

The clinical diagnosis of fibromyalgia (FM), a syndrome characterized by generalized pain, is challenging due to its unknown etiology and frequent comorbidity with other diseases. As a noninvasive modality, functional magnetic resonance imaging has been extensively employed in investigating the pathogenesis of FM. This study proposes a novel diagnostic approach utilizing resting-state functional magnetic resonance imaging (rs-fMRI) and diffusion tensor imaging (DTI) combined with a machine learning algorithm with the objective of enhancing the clinical diagnostic efficiency of FM. Two-sample t tests revealed differences between FM patients and healthy controls in rs-fMRI and DTI corresponding to brain image indices, mainly in the temporal lobe and frontal lobe. In addition, an effective diagnostic classification model was developed based on the single variable feature selection method by applying a support vector and random forest classifier combined with different brain image indicators. Our study demonstrated that the integration of DTI features with a support vector machine model yields superior diagnostic outcomes.

## Introduction

Fibromyalgia (FM) is a syndrome characterized by joint stiffness and chronic and generalized pain, primarily manifested as fatigue, sleep disturbances and tenderness in specific areas. It has a higher prevalence among females [1,2]. However, the pathogenesis of FM remains elusive. Currently, central sensitization is considered to be the primary mechanism, with genetic, immune and hormonal factors potentially playing pivotal roles [3,4]. FM, also referred to as the “prevalent comorbidity”, frequently manifests in conjunction with other diseases and remains concealed within common ailments [5,6]. The current status of FM diagnosis remains limited to clinical assessment [7], and dependable diagnostic markers are lacking. Due to these factors, the clinical detection of FM faces significant challenges, which are frequently associated with misdiagnosis.

Currently, neuroimaging techniques play a pivotal role in elucidating the pathogenesis of FM. Resting-state functional magnetic resonance imaging (rs-fMRI) could be used to investigate both the morphological and functional characteristics of brain regions in FM patients at rest based on the linear correspondence between the blood oxygenation level–dependent (BOLD) signal and neural activity [8]. During the onset of pain, patients with FM exhibit an augmented BOLD signaling response in the dorsolateral, ventrolateral, orbitofrontal cortex, frontal pole and precentral lobes [9]. Additionally, the brain network index is also an important rs-fMRI indicator, and another study reported that the brain connectivity between the insula and the default mode network was changed among patients with FM [10]. The research indicates that patients with FM demonstrate distinct functional abnormalities in interregional and global brain networks. However, there is still limited knowledge regarding the understanding of changes in functional network connectivity (FNC) among individuals with FM. Although static FNC (sFNC) is commonly employed for evaluating temporal correlations among brain regions, it fails to account for the potential variations in neurophysiological activities that individuals may demonstrate across diverse contexts and time periods [11]. Furthermore, increasing evidence highlights the complex and dynamic characteristics of the brain as a sophisticated system with temporal interdependencies [12,13]. Neuroimaging studies have demonstrated altered functional connectivity between the locus coeruleus (LC), hypothalamus, thalamus, and periaqueductal gray (PAG) in fibromyalgia (FM) patients [14]. Changes in dynamic functional network connectivity (dFNC) are associated with particular psychiatric disorders, cognitive states, and neurological ailments [15,16]. However, there is still a lack of research on the modifications in both sFNC and dFNC [17] among individuals diagnosed with FM.

In recent years, there has been a growing trend in both the research and application of diffusion tensor imaging (DTI), which utilize the anisotropy of water molecule diffusion to non-invasively display the microstructure of tissues such as white matter in the brain. This technique has been widely adopted by radiologists in routine clinical practice, with the majority of studies focusing on traumatic brain injury [18]. The diffusivity index of DTI typically encompasses the following four values: fractional anisotropy (FA), mean diffusivity (MD), radial diffusivity (RD) and axial diffusivity (AD) [19–21]. Based on DTI studies, patients with FM exhibited microstructural alterations in white matter connections within the medial corpus callosum, corpus callosum, perithalamic tracts, and connecting tracts [22]. Emotional pain, such as depressed mood, was found to be associated with FA values within the bilateral putamen and thalamus [22]. These imaging alterations observed in patients with FM offer the potential for identifying reliable imaging biomarkers for diagnostic purposes. However, the integration of image omics and machine learning in the clinical diagnosis of FM remains underutilized, with a focus only on the diagnostic efficacy of functional connectivity (FC) and structural features [23].

In this study, we combined machine learning with rs-fMRI and DTI data from the OpenNeuro database to construct a binary classification model of FM patients and healthy controls. The classification model comprises four distinct modules: data preprocessing, feature extraction, feature selection and classification. The feature extraction module systematically explored a vast array of original feature sets to identify altered brain regions in patients with FM, aiming to discover imaging markers that facilitate FM diagnosis. To achieve unbiased classification, the support vector machine (SVM) algorithm and random forest (RF) algorithm were used for classifying patients with FM and healthy controls, respectively. Finally, a comparison was made between these two models, resulting in a better classification model.

## Materials and methods

### Data sources

The rs-fMRI and DTI data were obtained from the OpenNeuro database (DS001928), with the complete acquisition parameters detailed in the original publication [24]. The data were categorized into four groups: precontrol, postcontrol, premusic and postmusic. In addition to the precontrol group, the remaining three groups of data were subjected to music or noise interference. The premusic group was collected 15 minutes after exposure to noise interference. Therefore, to minimize confounding factors, we selected the precontrol group for further analysis. All the subjects in this dataset are female.

### Data preprocessing of rs-fMRI data

The rs-fMRI data preprocessing in this study was conducted using the GRETNA (https://www.nitrc.org/projects/gretna) [25] toolbox of MATLAB 2020a and Advanced Normalization Tools (ANTs) (Version 2.3.1) [26] software. The specific steps were as follows: Initially, the first 5 fMRI images were excluded to mitigate instability. Second, time-level correction and head motion correction were performed, and subjects with head motion greater than or equal to 2 mm were excluded from the analysis. Two FM subjects were excluded due to failure to meet the inclusion criteria. Next, spatial standardization was performed using the ANTs to normalize the corrected fMRI images to a standardized space based on the Montreal Neurological Institute (MNI) Echo Planar Imaging (EPI) brain template. Finally, linear reduction and bandpass filtering (0.01–0.08 Hz) were used to mitigate low-frequency drift and attenuate high-frequency biological noise. Additionally, the generalized linear model was used to eliminate covariates associated with noise signals originating from head movement, white matter and cerebrospinal fluid. Ultimately, the analysis included 18 patients diagnosed with FM along with 20 healthy controls.

After completing the preprocessing stage, the data were mapped onto an anatomical automatic labeling (AAL) template using RESTplus (http://restfmri.net/forum/restplus) [27] toolbox. We selected and analyzed 90 brain regions from the AAL template and calculated brain network features, including graph theory and FC. Notably, Fisher-z transformation was used for FC conversion.

### Independent component analysis and FNC extraction

In this study, group spatial independent component analysis (ICA) was performed for 38 participants using the Group ICA of fMRI Toolbox (GIFT, https://trendscenter.org/software/gift), which by default applies spatial smoothing to individual-level component maps as part of its internal algorithmic workflow. In brief, spatial group ICA decomposition was performed using the Infomax algorithm to produce 40 independent components (ICs). According to the GIFT guidelines, 16 ICs were identified through visualization of components and validated by spatially matching them to pre-existing templates of 7 functional networks. Then, dynamic functional network connectivity (dFNC) was estimated as the Pearson correlation coefficients between pairs of time courses of the resting-state networks, resulting in a symmetric correlation matrix for each subject. Finally, correlations were transformed to z–scores using Fisher’s transformation to improve the normality for further statistical analyses.

### Functional network construction

We utilized the network analysis module of GRETNA software to conduct functional brain network analysis. The zFC data were input as a weighted undirected functional connection matrix, and the sparsity threshold was calculated to be 0.05:0.01:0.47. Subsequently, this threshold was applied to convert the matrix into a binary representation to mitigate the impact of pseudofunctional connections or weak functional connections. Within this threshold range, we calculated the global feature attribute, small-world property (*σ*), and local feature attribute, including the clustering coefficient (CC), shortest path length (SPL), nodal efficiency (NE), nodal local efficiency (NLE), degree centrality (DC) and betweenness centrality (BC), to describe the characteristics of the brain networks. A small-world network is a state of network that exhibits both local and global efficiency, bridging the gap between regular and random networks while incorporating their respective topological advantages.

### Preprocessing of DTI data

The FSL (http://www.fmrib.ox.ac.uk/fsl) tool was used in this study to preprocess the DTI data. First, the original diffusion-weighted images were corrected for head motion and eddy currents (eddy_correct), and the gradient directions were simultaneously rotated. Then, the brain mask was extracted from the B0 image. The diffusion tensor fitting was performed using the dtifit tool to generate fractional anisotropy (FA) and mean diffusivity (MD) maps. The axial diffusivity (AD) map was obtained from the principal eigenvalue map (L1), and the radial diffusivity (RD) map was calculated by averaging the secondary eigenvalues (L2 and L3). To achieve inter-group comparability, the analysis was conducted in a standardized spatial framework. First, each subject’s FA image was registered to their T1 structural image, and then further registered to the standard template space. Subsequently, the standard AAL atlas was registered to each subject’s original diffusion space through inverse transformation. Finally, within this individual space, the average index values of 90 brain regions were extracted from the FA, MD, AD, and RD maps using the registered atlas for subsequent analysis.

### Statistical analysis

The SPM 12, BrainNet [28], NBS [29], GRETNA toolboxes of MATLAB 2020a and R were used for statistical analysis. For the comparison of imaging omics features between the FM group and the healthy control group. (1) FC and graph theory features employed two-sample. Multiple comparisons were corrected using the Network-Based Statistic (NBS) approach, with the statistical significance threshold set at a family-wise error (FWE) corrected P < 0.05. (2) DTI features (FA, MD, AD, RD conducted two-sample t-test for each metric. The resulting *P*-values were corrected for multiple comparisons across brain regions using the Benjamini-Hochberg (BH) false discovery rate (FDR) method. The statistical significance was defined as *q* < 0.05.

The resulting connectivity networks from the NBS analysis were visualized using BrainNet Viewer.

### Feature selection

We acquired a substantial amount of feature data from rs-fMRI data. However, due to the computational constraints associated with high-dimensional data, the incorporation of numerous features into machine learning analysis may result in overfitting issues. Therefore, prior to classification, it is imperative to perform feature selection as a crucial step in the research process. The achievement of dimensionality reduction lies in the selection of appropriate features and the filtration of unimportant or irrelevant features, which not only reduces the time required for training and testing datasets but also enhances the classification accuracy. The univariate feature selection method used in this study involved scoring and ranking each feature based on its performance in a univariate statistical test, followed by the selection of the best feature. The selectKBest function provided by the scikit-learn library’s feature_selection module in Python (Version 3.13.3) was utilized to perform univariate feature selection. A parameter k = 10 was set, and the top 10 features with the highest scores were chosen as the final set of feature variables.

### Support vector machine

SVM is a machine learning algorithm commonly used for addressing binary classification problems. The SVM classifier was implemented using the scikit-learn library in Python. To optimize the classification performance, a grid search and 10-fold cross-validation were used for parameter tuning of C, kernel and gamma. The 10-fold cross-validation method is commonly used to evaluate the algorithm’s accuracy. The dataset is partitioned into 10 subsets, with 9 of them allocated for training purposes and one reserved as a test subset for verification. After dividing the dataset into two mutually exclusive parts, namely, the training set and the test set, at a 7:3 ratio, we further partitioned the training set into 10 equally sized subsets prior to conducting 10-fold cross-validation. For each iteration, 9 subsets are chosen as the training set, while the remaining subset is utilized for validation, resulting in a total of 10 models. The classification rate is determined by evaluating the performance of the 10 models on the test set and subsequently calculating the average of these rates as the final classification rate. This methodology has also been used by RF classifiers for validation purposes.

### Random forest

RF is an ensemble learning technique that can be applied to both classification and regression problems. The parameters for RF adjustment include those for the RF framework and decision tree. The n_estimator parameter of the RF framework and the max_depth parameter of the RF decision tree are optimized in our model. N_estimators, known as the number of decision trees, corresponds to the number of subdatasets in the original dataset. An insufficient number of n_estimators may result in underfitting, whereas an excessive number does not significantly enhance the model’s performance. Max_depth is the maximum depth of the decision tree. The RF classifier utilized in this study was implemented through the scikit-learn library.

## Results

### Clinical and demographic characteristics

A total of 18 FM patients and 20 healthy controls were included in this study to investigate the neuroimaging mechanisms underlying alterations in FM. The clinical and demographic information of the subjects is presented in Table 1. The results indicated that there was no statistically significant difference in age between the FM and healthy control groups under study, thereby mitigating the potential influence of age on the selection of imaging features. Furthermore, Student’s t test revealed that FM patients exhibited significant increases in pain catastrophizing scale (PCS), state-trait anxiety inventory (STAI) and Center for Epidemiologic Studies Depression Scale (CES–D) scores compared to healthy controls, consistent with previous behavioral studies on FM [30,31].

**Table 1:** Demographic and clinical characteristics.

Demographic information	FM (n = 18) Mean ± SD	HC (n = 20) Mean ± SD	*P–*value
**Age (year)**	43.94 ± 10.56	42.10 ± 12.49	0.625
**Process (year)**	4.86 ± 4.44	/	/
**PCS**	26.61 ± 12.77	12.00 ± 10.93	< 0.001
**STAI**	52.11 ± 20.98	26.05 ± 10.66	< 0.001
**CES–D**	29.94 ± 14.34	11.00 ± 8.64	< 0.001

FM, fibromyalgia; HC, healthy control; PCS, Pain Catastrophizing Scale; STAI, State-Trait Anxiety Inventory; CES-D, Center for Epidemiologic Studies Depression Scale.

### Changes in static brain network connectivity

**Fig 1:**
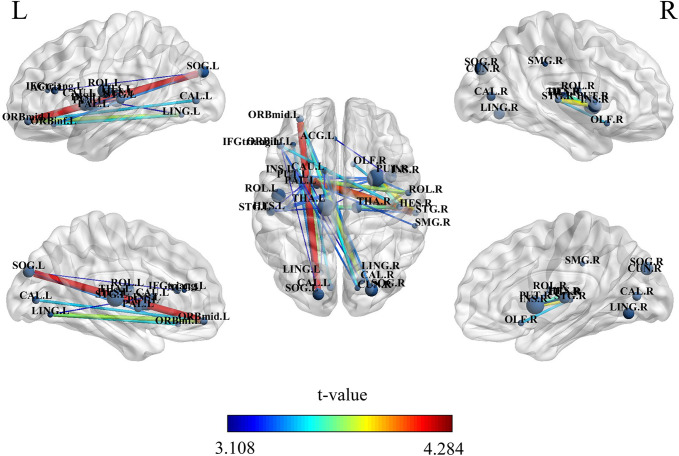
Functional connectivity matrix area mapping of the subject during the resting state. Compared with that in the healthy control group, the FC in the FM group was altered. MFGorb, medial orbital of the superior frontal gyrus; CAL, calcarine fissure and surrounding cortex; IFGtri, triangular part of the inferior frontal gyrus; CUN, cuneus; LING, lingual gyrus; SOG, superior occipital gyrus; INS, insula; ACC, anterior cingulate and paracingulate gyri; CAU, caudate nucleus; ROL, rolandic operculum; PUT, lenticular nucleus, putamen; PAL, lenticular nucleus, pallidum; SMG, supramarginal gyrus; THA, thalamus; HES, Heschl’s gyrus; STG, superior temporal gyrus; OLF, olfactory cortex; CAU, caudate nucleus.

To investigate alterations in sFNC features between patients with FM and healthy controls, we compared FC and graph theory features between the two groups, revealing disparities in static brain network features between the two cohorts. Compared with that in the healthy control group, the FC in the FM group was altered (Fig 1 and S1 Table). NBS analysis revealed a single connected component with 27 nodes and 44 connections that showed lower functional connectivity (*P* = 0.037 corrected) in FM patients. Weakened connectivity within this single component was mainly located in the temporal lobe, which includes the right and left superior temporal gyrus (STG), and the frontal lobe, which includes the left medial orbital of the superior frontal gyrus (MFGorb), triangular part of the inferior frontal gyrus (IFGtri) and inferior frontal gyrus pars orbitalis (IFGorb). These findings suggest that temporal lobe and frontal lobe regions have decreased resting brain connectivity and that their decreased connectivity is associated with each other as part of a single, integrated abnormal brain circuitry in FM.

**Fig 2:**
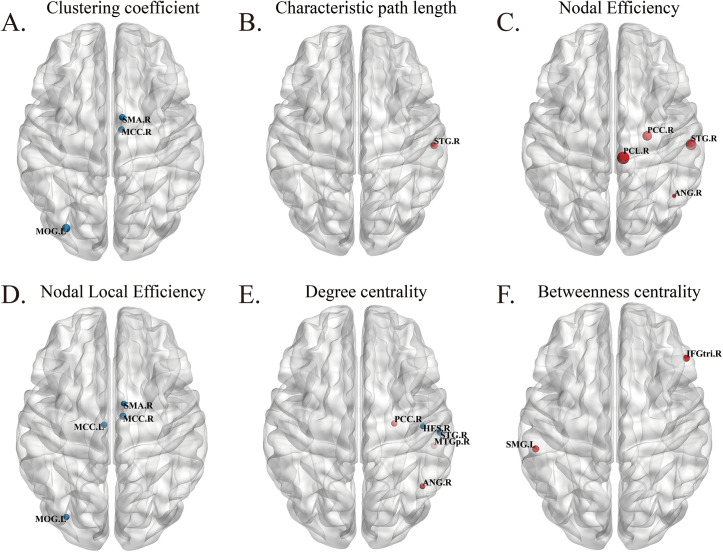
Group differences in graph theory features. (A) Regions with significant between-group differences in the clustering coefficient; (B) Brain regions with differences in characteristic path length; (C) Brain regions with differences in nodal efficiency; (D) Brain regions with differences in nodal local efficiency; (E) Brain regions with differences in degree centrality; (F) Brain regions with differences in betweenness centrality. SMA: supplementary motor area; MCC, middle cingulate cortex; MOG, middle occipital gyrus; STG, superior temporal gyrus; PCC, posterior cingulate cortex; ANG, angular gyrus; PCL, paracentral lobule; HES, Heschl’s gyrus; MTGp, middle temporal gyrus of temporal pole; IFGtri, triangular par of inferior frontal gyrus; SMG, supramarginal gyrus. The red nodes represent the regions showing abnormally high nodal properties in the FM, and the blue nodes represent regions showing lower nodal properties in the FM than in the healthy controls (*P* < 0.05). Red, increased; Blue, decreased.

To further investigate the changes in network features, this study used FC using the GRETNA toolbox to compute various graph theory indices of the static functional network. Both FM patients and HCs exhibited *σ* (*σ* > 1) in the entire range of connectivity density within their respective brain networks, with a significant difference between them (Fig 2 and S2 Table). Compared to the healthy control group, the FM group exhibited significant reductions in CC within the right supplementary motor area (SMA), right middle cingulate cortex (MCC) and left middle occipital gyrus (MOG) (S1A Fig). The SPL exhibited a significant increase in the right superior temporal gyrus (STG) (S1B Fig). The levels of NE were significantly increased in the right posterior cingulate cortex (PCC), right angular gyrus (ANG) and right paracentral lobule (PCL), while they were significantly decreased in the right STG (S2A Fig). The NLEs in the right SMA, right and left MCC and left MOG significantly decreased (S2B Fig). There was a significant decrease in DC in the right Heschl’s gyrus (HES) and right STG in individuals with FM. Conversely, there was a significant increase in DC in the right PCC, right ANG and right middle temporal gyrus of the temporal pole (MTGp) (S3A Fig). BC exhibited a significant increase in the right IFGtri, as well as in the left supramarginal gyrus (SMG) (S3B Fig). In summary, compared to those in healthy controls, alterations in the brain network features of FM patients predominantly manifested in the temporal lobe and frontal lobe. The right STG also exhibited changes across multiple graph theory metrics.

### Changes in dynamic brain network connectivity

**Fig 3:**
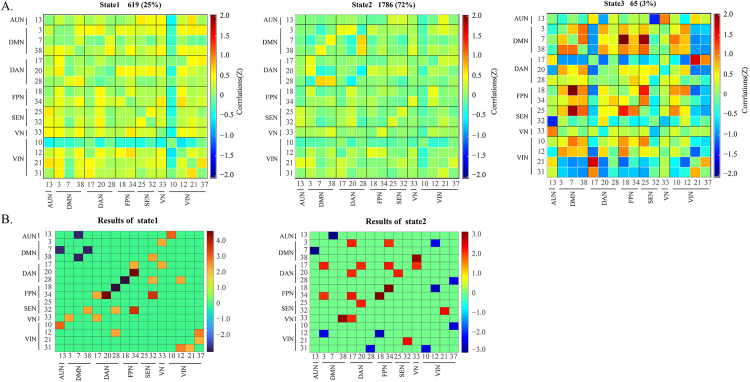
Four distinct functional states identified by k-means cluster analysis and corresponding cluster centroids. (A) Group centroid matrices for each state. (B) Matrix plot of significant dynamic functional network connectivity differences between the fibromyalgia and healthy control groups (*P* < 0.05). VIN, visual network; SEN, sensory–motor network; AUN, auditory network; DAN, dorsal attention network; VAN, ventral attention network; FPN, frontoparietal network; DMN, default mode network. Red, increased; Blue, decreased.

Using GIG–ICA decomposition, 40 ICA components were obtained, from which 16 ICA components were selected as ICNs. Based on anatomical and functional characteristics, 16 ICA further divided into 7 networks including the visual network (VIN), sensory–motor network (SEN), auditory network (AUN), dorsal attention network (DAN), ventral attention network (VAN), frontoparietal network (FPN), and default mode network (DMN). K-means clustering was used to determine the three recurring dynamic functional network connectivity (dFNC) states over time (Fig 3A). State 1 (accounting for 25% of all time windows) showed increased or decreased FC within and between certain networks, with the VIN’s connections to other functional networks generally weakened. State 2 (accounting for 72% of all time windows) had sparse connections and showed a general weakening of functional network connections throughout the brain. State 3 (accounting for 3% of all time windows) was characterized by modular connections, with the DMN and FPN showing a clear positive module connection to the SEN. Independent sample t-tests were used to further compare the dFNC matrices of each state between the groups, and it was found that the dFNC of FM patients was significantly different from that of HC in states 1–2 (Fig 3B). In state 1, the weakened brain network connections for dFNC were DMN–AUN and FPN–DAN; the increased brain network connections for dFNC were VIN–AUN, FPN–DAN, and SEN–FPN, with the highest connection strength between PN and DAN. In state 2, the weakened brain network connections for dFNC were DMN–AUN, DMN–VIN, and DAN–VIN, with the lowest connection strength between DMN and AUN; the increased brain network connections included DAN–DMN, DAN–FPN, FPN–DMN, VN–DMN, VN–DAN, and VIN–SEN, with the highest connection strength between VN–DMN.

**Fig 4:**
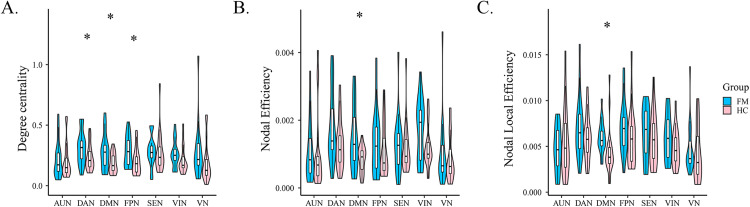
Comparisons of nodal properties of dynamic functional network connectivity. (A) Differences in the degree centrality of dFNC among FM and HC. (B) Differences in the nodal efficiency of dFNC among FM and HC. (C) Differences in the nodal local efficiency of dFNC among FM and HC. FM, fibromyalgia; HC, healthy control. VIN, visual network; SEN, sensory–motor network; AUN, auditory network; DAN, dorsal attention network; VAN, ventral attention network; FPN, frontoparietal network; DMN, default mode network. *P < 0.05.

In addition, three important graph theory indices were determined in this study: DC, NE, and NEL (*P* < 0.05) (Fig 4). The DC values of DAN and FPN in the FM group were higher than those in the HC group. The DC, NE, and NLE values of the DMN in all FM groups were higher.

### Changes in diffusion tensor imaging

**Fig 5:**
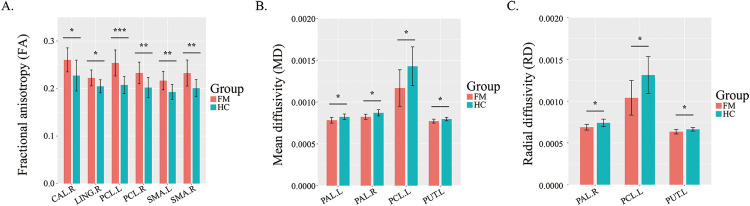
Brain regions with altered DTI characteristics in FM patients compared with healthy controls. (A) The brain regions exhibiting notable disparities in FA. (B) The brain regions exhibiting notable disparities in MD. (C) The brain regions exhibiting notable disparities in AD patients. (D) The brain regions exhibiting notable disparities in RD patients. DTI, diffusion tensor imaging; FM, fibromyalgia; HC, healthy control; SMA, supplementary motor area; CAL, calcarine fissure and surrounding cortex; LING, lingual gyrus; PCL, paracentral lobule; PUT, putamen; PAL, pallidum. *q < 0.05, **q < 0.01, ***q < 0.001.

To explore the changes in brain white matter fibers in FM patients, four indices of DTI image data—FA, MD, AD and RD—were calculated in this paper, and the results were compared with those in the healthy control group through two-sample t tests (S3 Table and Fig 5). The brain regions with significantly greater FA values in FM patients than in HCs included the right and left SMA, right CAL, right LING, and right and left PCL. The brain regions with significantly lower MD values included the left PCL, right PUT and left and right PAL. An increase in the FA value and a decrease in the MD value reflected an increase in the degree of myelination of white matter fibers. Interestingly, the brain regions with significant changes in RD values were consistent with those with significant changes in MD values, except for the right-sided CAL.

### Construction of a machine learning model based on image omics features

The combination of brain network features and machine learning models was used to evaluate the application of imaging indicators in diagnosing FM patients. The classification performances of the machine learning models were compared under combinations of FC features (feature group 1), graph theory features (feature group 2), ICA features (feature group 3), and DTI features (feature group 4). The model performance was comprehensively evaluated using multiple indicators such as area under the curve (AUC), accuracy rate (ACC), true positive rate (TPR), true negative rate (TNR), false positive rate (FPR), and F1 score (Table 2).

**Table 2:** Classification efficiency of different combinations of features combined with machine learning.

Machine learning	Features group	AUC ± SD	ACC ± SD	TPR ± SD	TNR ± SD	FPR ± SD	F1 score ± SD
**SVM**	FC	0.79 ± 0.15	0.67 ± 0.12	0.62 ± 0.19	0.72 ± 0.19	0.67 ± 0.21	0.62 ± 0.14
	Graph theory	0.90 ± 0.29	0.68 ± 0.04	0.71 ± 0.27	0.80 ± 0.19	0.71 ± 0.28	0.71 ± 0.15
	Graph theory + FC	0.87 ± 0.23	0.72 ± 0.42	0.68 ± 0.21	0.84 ± 0.16	0.68 ± 0.28	0.68 ± 0.18
	ICA	0.75 ± 0.08	0.67 ± 0.06	0.78 ± 0.17	0.64 ± 0.23	0.78 ± 0.21	0.78 ± 0.11
	DTI	0.98 ± 0.02	0.83 ± 0.35	0.92 ± 0.14	0.85 ± 0.10	0.92 ± 0.12	0.92 ± 0.09
**RF**	FC	0.63 ± 0.83	0.60 ± 0.08	0.60 ± 0.13	0.67 ± 0.20	0.61 ± 0.13	0.56 ± 0.07
	Graph theory	0.84 ± 0.09	0.74 ± 0.06	0.74 ± 0.15	0.84 ± 0.20	0.71 ± 0.19	0.65 ± 0.09
	Graph theory + FC	0.83 ± 0.07	0.65 ± 0.06	0.65 ± 0.16	0.80 ± 0.15	0.65 ± 0.13	0.66 ± 0.11
	ICA	0.87 ± 0.12	0.65 ± 0.08	0.65 ± 0.18	0.80 ± 0.17	0.65 ± 0.17	0.65 ± 0.16
	DTI	0.90 ± 0.04	0.79 ± 0.08	0.79 ± 0.14	0.84 ± 0.11	0.74 ± 0.09	0.75 ± 0.09

AUC, area under the curve; ACC, accuracy; FC, functional connectivity; DTI, diffusion tensor imaging; ICA, Independent component analysis; SVM, support vector machine; RF, random forest.

**Fig 6:**
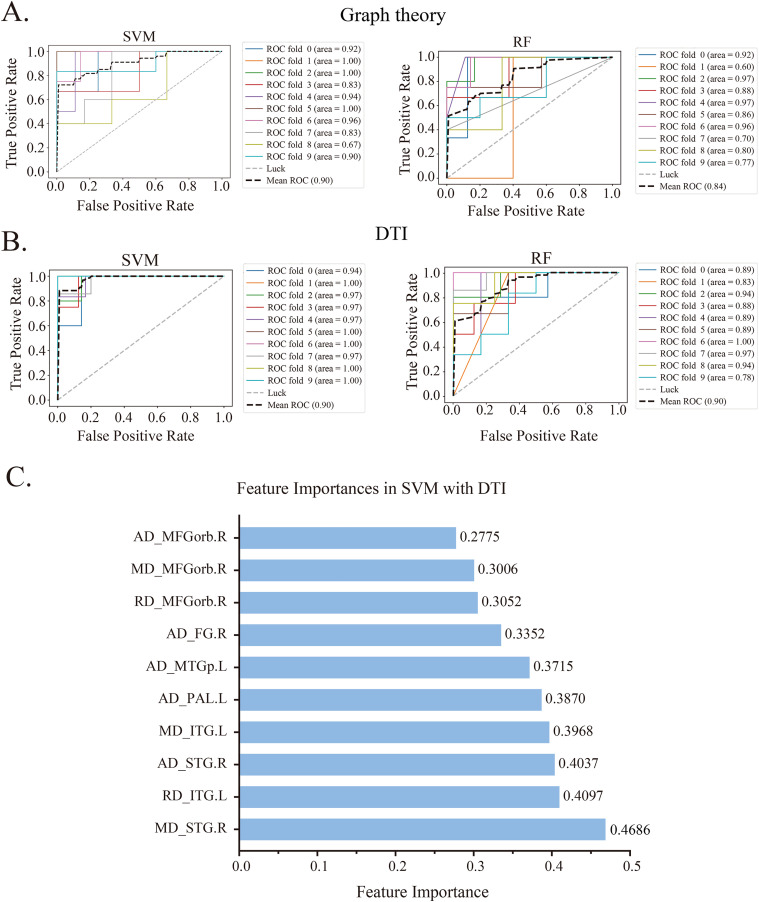
The classification efficiency of imaging features combined with machine learning, as indicated by the receiver operating characteristic (ROC) curve. (A) Diagnostic classification efficiency of graph theory features; (B) Diagnostic classification efficiency of DTI features. (C) The importance of the 10 features in the diagnostic classification model constructed by combining DTI features with SVM. AUC, area under the curve; DTI, diffusion tensor imaging; SVM, support vector machine; RF, random forest; AD, axial diffusivity; MD, mean diffusivity; RD, radial diffusivity; MFGorb, medial orbital of the superior frontal gyrus; FG, Fusiform gyrus; MTGp, middle temporal gyrus of temporal pole; PAL, lenticular nucleus, pallidum; ITG, Inferior temporal gyrus; STG, superior temporal gyrus.

Given the abundance of original imaging features obtained in this study, we used univariate feature selection as a means of reducing dimensionality to mitigate potential overfitting issues. Finally, the top 10 most significant features in each of the three combinations were identified for the diagnosis of FM patients (Fig 6 A and 6B; Table 2 and S4A and S4B Fig). The efficiency of the SVM model: the AUC values for prediction using feature groups 1, 2, 3, and 4 were 0.79, 0.90, 0.75, and 0.98, respectively. In terms of the efficiency of the RF model, the AUC values for prediction using feature groups 1, 2, 3, and 4 were 0.63, 0.84, 0.87, and 0.90, respectively. Among the static brain network connectivities, the graph theory features had the best classification effect. Among the 10 imaging feature indicators in this model, the MD value of the right STG showed the highest contribution (Fig 6C). This finding suggests that investigating STG alterations may provide new insights into FM research.

## Discussion

Early diagnosis and treatment of FM can effectively alleviate pain and improve the quality of life of patients. Currently, the increasing application of machine learning in clinical diagnosis, particularly in conjunction with imaging techniques, is facilitating the construction of novel diagnostic models. As a result, the combination of imaging biomarkers and machine learning has promising potential in the clinical diagnosis and evaluation of FM.

The present study combined sFNC and dFNC analyses to investigate the whole brain features of FM. The analysis of sFNC revealed that the disparities in the brain network features of FM predominantly manifested in the temporal lobe and lobus frontalis. The connections between several brain regions of FM patients were attenuated, with the STG serving as the central hub. Moreover, the SPL and DC of the STG in the FM were reduced, while the NE was increased. The MD index of STG plays an important role in the construction of diagnostic classification models. The STG in the temporal lobe is widely recognized as the primary auditory cortical center. Previous studies have shown that FM patients have increased sensitivity to normal and harmless external stimuli, including auditory and visual stimuli [32–35], which may arise from alterations in brain networks associated with the STG. Frontal lobe activity during pain is commonly associated with attentional processing in the context of nociceptive stimuli [36]. The prefrontal cortex and orbitofrontal cortex play pivotal roles in the cognitive modulation of pain. Herein, the connectivity of the brain network centered on the left MFGorb and IFGtri was reduced in patients with FM. These results suggest that FM may lead to cognitive impairments among patients.

In comparing dFNC differences between groups, we found the results were highly similar to those of sFNC, with subtle differences between the different states, which mainly reflected in DMN. Previous studies have shown that DMN mainly includes the PCC, medial prefrontal cortex, inferior parietal lobule, and bilateral temporal cortex. Therefore, our results suggest that FM should be associated with changes in temporal and frontal brain functional features. Furthermore, previous studies have shown that positive coping strategies may reduce the degree of change in the DMN, while negative coping styles (such as catastrophizing thinking) may aggravate the change in the DMN [37]. This indicates that the changes of DMN are not only related to the pain itself, but also closely related to the patients’ cognition of pain and emotional regulation. In state 1, the dFNC value between DMN and AUN decreases, and in state 2, DMN and AUN also decline. The AUN includes the primary auditory cortex and the secondary auditory cortex. The primary auditory cortex mainly encodes the characteristics of auditory stimuli, while the secondary auditory cortex integrates and connects this information to generate specific perceptions [38]. Patients with FM often have emotional problems such as anxiety and depression. The weakened connection between DMN and AUN indicates that the external perception ability and self-awareness integration of FM patients are impaired, which may lead to further intensification of pain.

In this study, we employed the comprehensive AAL atlas to explore functional connectivity across all brain regions, rather than limiting our analysis to previously established pain-related regions of interest (ROI). This approach allowed us to identify significant changes in regions such as the STG and frontal areas, which are not classically considered core pain regions but appear functionally and structurally involved in FM pathology, possibly related to auditory processing, cognitive modulation, and central sensitization. However, the inclusion of all regions also introduces challenges in interpretability and specificity, as it may increase the dimensionality of the data and incorporate noise from unaffected areas. To mitigate this, we employed machine learning models capable of handling high-dimensional data and applied rigorous cross-validation to ensure that identified features were robust. Future studies could benefit from combining hypothesis-driven ROI analyses with exploratory whole-brain approaches to balance specificity with the discovery of novel biomarkers.

DTI analysis revealed an apparent increase in fiber myelination levels in FM patients’ brains, primarily evidenced by elevated FA values across multiple brain regions, consistent with previous studies [39,40]. The PCL is located on the inner side of the cerebral hemisphere and is mainly involved in motor and sensory functions, especially the motor and sensory control of the lower limbs. The white matter fibers of the PCL are mainly responsible for transmitting neural signals related to lower extremity movement and sensation. SMA is located in the prefrontal lobe of the brain and is related to motor control and planning. The white matter fibers of SMA are mainly involved in the preparation and execution processes of sports. The increase of FA values in PCL and SMA may suggest that the microstructure of white matter fibers has changed, such as increased fiber density, improved myelination degree, or more orderly fiber arrangement. Among the graph theory indicators, the decrease of CC value and NLE value of SMA, and the increase of NE value of PCL also suggest the changes in the brain network functions of SMA and PCL. However, there is no direct study yet to clarify the relationship between the elevated FA values of PCL and SMA and fibromyalgia. Central sensitization in patients with fibromyalgia may lead to changes in the microstructure of white matter fibers in the brain [41], thereby affecting the transmission of neural signals. The elevated FA values of PCL and SMA may be a manifestation of white matter fiber changes caused by neuroinflammation, and such changes may be related to the pain and motor dysfunction of fibromyalgia. However, there is limited research on DTI in the field of FM, and variations observed in the specific brain regions exhibiting changes in FA values necessitate further investigation for conclusive results.

Numerous studies have consistently demonstrated that structural and functional magnetic resonance imaging can effectively identify changes in specific features (either in structure or function) within patient cohorts, serving as valuable biological indicators for associated diseases [42,43]. In this study, the diagnostic classification model constructed by combining SVM with imaging indicators has a better effect. This might be because SVM performs better when dealing with sparse data and high-dimensional data. It maps the data to the high-dimensional space through kernel techniques and looks for the optimal segmentation hyperplane in the high-dimensional space, thereby being able to handle linearly inseparable data very well. The optimization objective of SVM is to maximize the margin. It mainly relies on support vectors, that is, the few points closest to the classification boundary. Therefore, it has a strong fitting ability for small sample data. When the amount of data is small, SVM can prevent overfitting by adjusting the regularization parameters, such as the C value. While RF builds multiple decision trees through bootstrap sampling, when the sample size is small, self-sampling may lead to insufficient sample diversity, thereby affecting the generalization ability of the model. Furthermore, the image markers identified through machine learning models were not significantly different (S4 and S5 Tables). This intriguing phenomenon has been frequently observed in other neuroimaging studies [44]. However, our diagnostic model, which integrates DTI features and the SVM model, achieved an AUC of 98% in this study. The greater property of DTI compared to rs-fMRI suggests its diagnostic potential for FM. Since DTI is primarily used for visualizing and quantifying the integrity of white matter, the above results revealed that the changes in microstructural complexity of FM were more prominent in white matter than in gray matter, which indicates that investigating alterations in white matter networks in FM could offer valuable insights into the progression of this disease.

Nevertheless, this study still has certain limitations that require further refinement. First, the limited sample size results in a machine learning model that still has considerable training space to improve, which partly impacts the predictive capability of the classification model. Additionally, the imaging data used in this study were derived from a single source; therefore, it is better to obtain a substantial number of samples from diverse sources to validate the robustness of our results. To assess model performance under our limited sample size, we employed a 10-fold cross-validation scheme. While this approach provides a robust performance estimate, we acknowledge that the risk of overfitting remains a significant concern in small-sample studies. Future multicenter studies will employ larger cohorts and multimodal data integration to validate model robustness, while functional neuroimaging experiments will characterize fibromyalgia-associated brain network reorganization.

## Conclusion

This study provides evidence suggesting that cognitive network alterations in FM patients may be associated with microstructural changes in the STG and frontal regions, along with increased complexity of brain tissue organization. Our preliminary findings indicate that combining DTI features with machine learning could offer a promising direction for developing diagnostic tools. Future studies with larger sample sizes, additional imaging modalities, and optimized feature selection are needed to validate and extend these findings toward clinical application.

## Supporting information

S1 Fig The AUC values of clustering coefficient (CC) and shortest path length (SPL). The AUC values of graph theory features were compared, along with the variation trend under the sparse threshold ranging from 0.05 to 0.47. (A) Brain regions with differences in clustering coefficient; (B) Brain regions with differences in shortest path length. AUC, the area under the curve; FM, fibromyalgia; HC, healthy controls; SMA: supplementary motor area; MCC, middle cingulate cortex; MOG, middle occipital gyrus; STG, superior temporal gyrus.
(PDF)

S2 Fig The AUC values of nodal efficiency (NE) and nodal local efficiency (NLE). The AUC values of graph theory features were compared, along with the variation trend under the sparse threshold ranging from 0.05 to 0.47. (A) Brain regions with differences in nodal efficiency; (B) Brain regions with differences in nodal local efficiency. AUC, the area under the curve; FM, fibromyalgia; HC, healthy controls; PCC, posterior cingulate cortex; ANG, Angular gyrus; PCL, paracentral lobule; STG, superior temporal gyrus.
(PDF)

S3 Fig The AUC values of degree centrality (DC) and betweenness centrality (BC). The AUC values of graph theory features were compared, along with the variation trend under the sparse threshold ranging from 0.05 to 0.47. (A) Brain regions with differences in degree centrality; (B) Brain regions with differences in betweenness centrality. AUC, the area under the curve; FM, fibromyalgia; HC, healthy controls; SMA: supplementary motor area; MCC, middle cingulate cortex; MOG, middle occipital gyrus; PCC, posterior cingulate cortex; ANG, Angular gyrus; HES, heschl’s gyrus;; STG, superior temporal gyrus; MTGp, middle temporal gyrus of temporal pole; IFGtri, triangular par of inferior frontal gyrus; SMG, supramarginal gyrus.
(PDF)

S4 Fig The classification efficiency of machine learning. The classification efficiency of imaging features combined with machine learning, as indicated by AUC. (A) Diagnostic classification efficiency of functional connectivity features; (B) Diagnostic classification efficiency of graph theory and functional connectivity features; (C) Diagnostic classification efficiency of ICA. AUC, the area under the curve; ACC, accuracy; FC, functional connectivity; DTI, diffusion tensor imaging; ICA, independent component analysis; SVM, support vector machine; RF, random forest.
(PDF)

S1 Table Functional connectivity with significant group differences. MFGorb, medial orbital of superior frontal gyrus; CAL, calcarine fissure and surrounding cortex; IFGtri, triangular part of inferior frontal gyrus; CUN, cuneus; LING, lingual gyrus; SOG, superior occipital gyrus; INS, insula; ACC, anterior cingulate and paracingulate gyri; CAU, caudate nucleus; ROL, rolandic operculum; PUT, lenticular nucleus, putamen; PAL, lenticular nucleus, pallidum; SMG, supramarginal gyrus; THA, thalamus; HES, Heschl’s gyrus; STG, superior temporal gyrus; OLF, olfactory cortex; CAU, caudate nucleus.
(PDF)

S2 Table Brain regions exhibiting altered graph theory features. SMA: supplementary motor area; MCC, middle cingulate cortex; MOG, middle occipital gyrus; STG, superior temporal gyrus; PCC, posterior cingulate cortex; ANG, Angular gyrus; PCL, paracentral lobule; HES, heschl’s gyrus; MTGp, middle temporal gyrus of temporal pole; IFGtri, triangular par of inferior frontal gyrus; SMG, supramarginal gyrus.
(PDF)

S3 Table Brain regions with changed DTI features. DTI, diffusion tensor imaging; SMA, supplementary motor area; CAL, calcarine fissure and surrounding cortex; LING, lingual gyrus; PCL, paracentral lobule; PUT, putamen; PAL, pallidum.
(PDF)

S4 Table The graph theory features were obtained through single variable feature selection screening. SMA, supplementary motor area; MCC, middle cingulate cortex; MOG, middle occipital gyrus; PCC, posterior cingulate cortex; ANG, angular gyrus; STG, superior temporal gyrus; IFGtri, triangular par of inferior frontal gyrus; SMG, supramarginal gyrus.
(PDF)

S5 Table DTI features obtained from univariate feature selection screening. DTI, diffusion tensor imaging; MFG, middle frontal gyrus; FG, fusiform gyrus; PAL, pallidum; STG, superior temporal gyrus; MTGp, middle temporal gyrus of temporal pole; ITG, inferior temporal gyrus.
(PDF)
