# Supplementary figures and images for: Machine learning for the diagnosis of fibromyalgia based on magnetic resonance imaging
Source: PLoS One. 2026 Feb 2;21(2):e0340899. doi: 10.1371/journal.pone.0340899 (PMC12863509; doi:10.1371/journal.pone.0340899)

A.

### Degree centrality

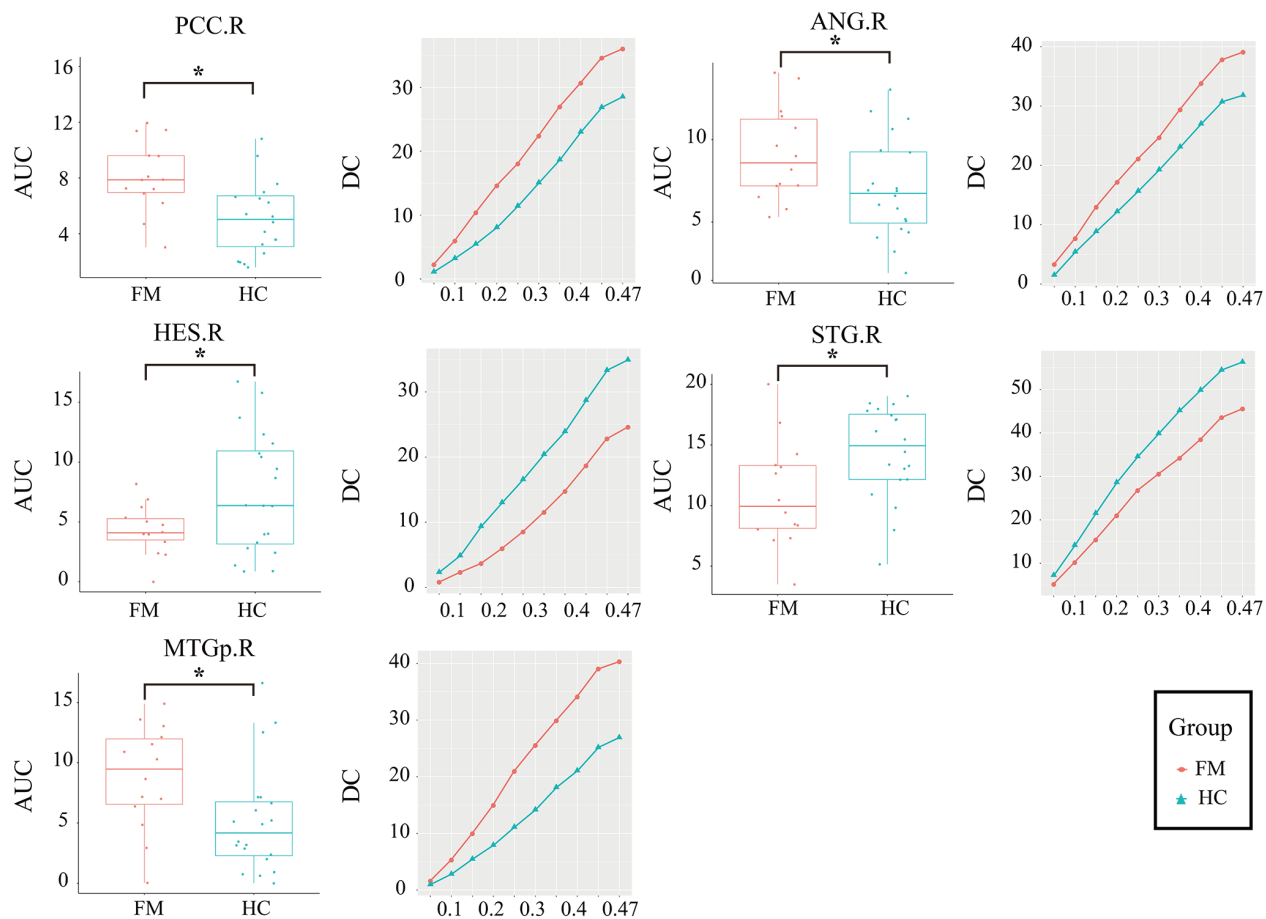

B.

### Betweenness centrality

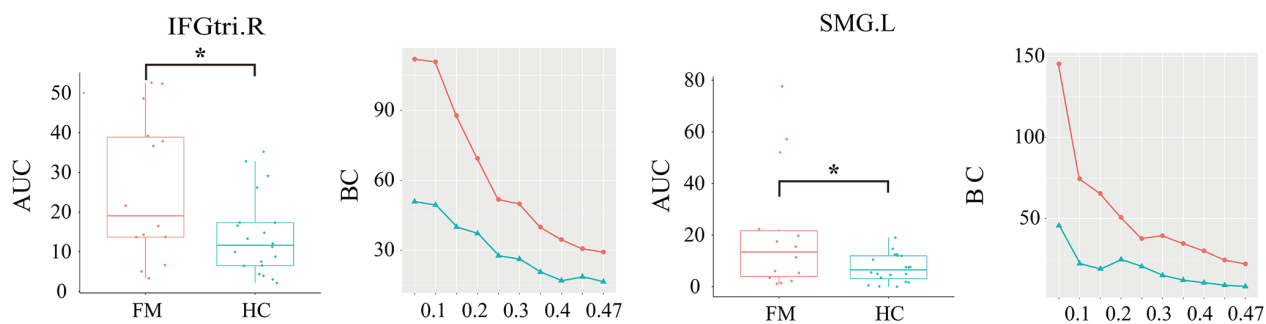

Supplement: S1 Fig — The AUC values of graph theory features were compared, along with the variation trend under the sparse threshold ranging from 0.05 to 0.47. (A) Brain regions with differences in clustering coefficient; (B) Brain regions with differences in shortest path length. AUC, the area under the curve; FM, fibromyalgia; HC, healthy controls; SMA: supplementary motor area; MCC, middle cingulate cortex; MOG, middle occipital gyrus; STG, superior temporal gyrus. (PDF) [file pone.0340899.s001.pdf]

A.

## Nodal Efficiency

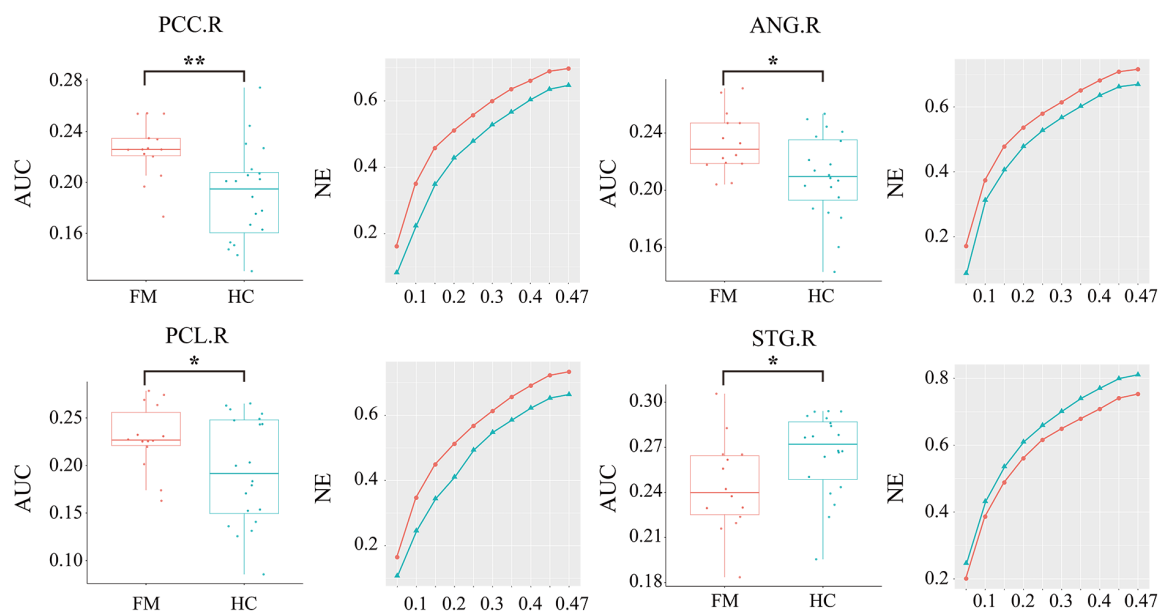

B.

## Nodal Local Efficiency

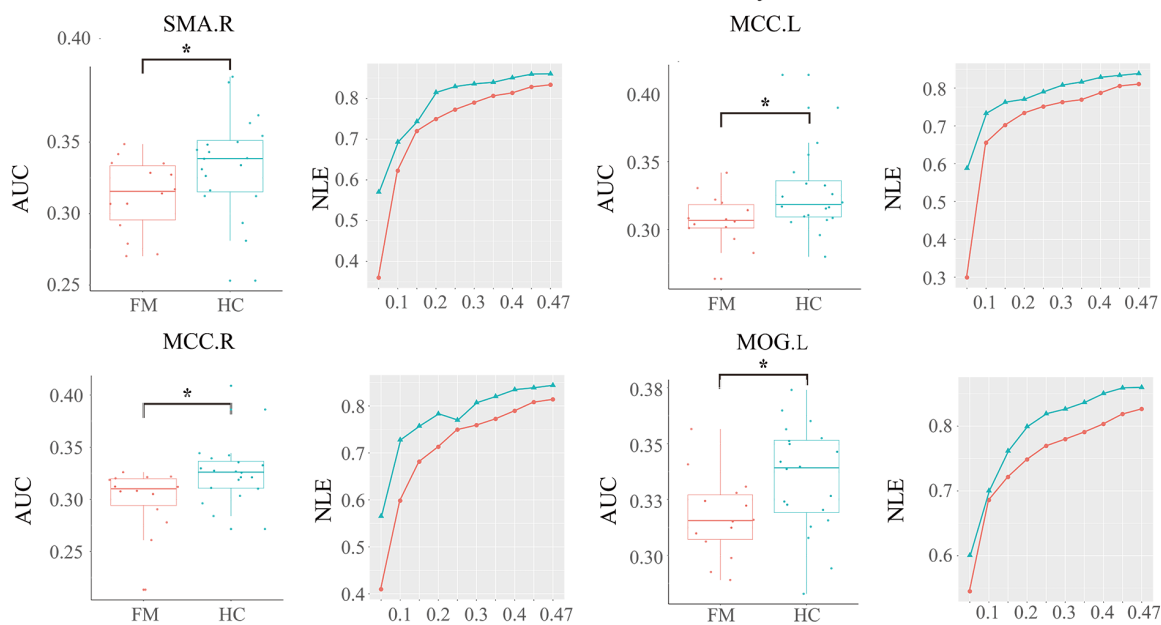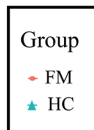

Supplement: S2 Fig — The AUC values of graph theory features were compared, along with the variation trend under the sparse threshold ranging from 0.05 to 0.47. (A) Brain regions with differences in nodal efficiency; (B) Brain regions with differences in nodal local efficiency. AUC, the area under the curve; FM, fibromyalgia; HC, healthy controls; PCC, posterior cingulate cortex; ANG, Angular gyrus; PCL, paracentral lobule; STG, superior temporal gyrus. (PDF) [file pone.0340899.s002.pdf]

A.

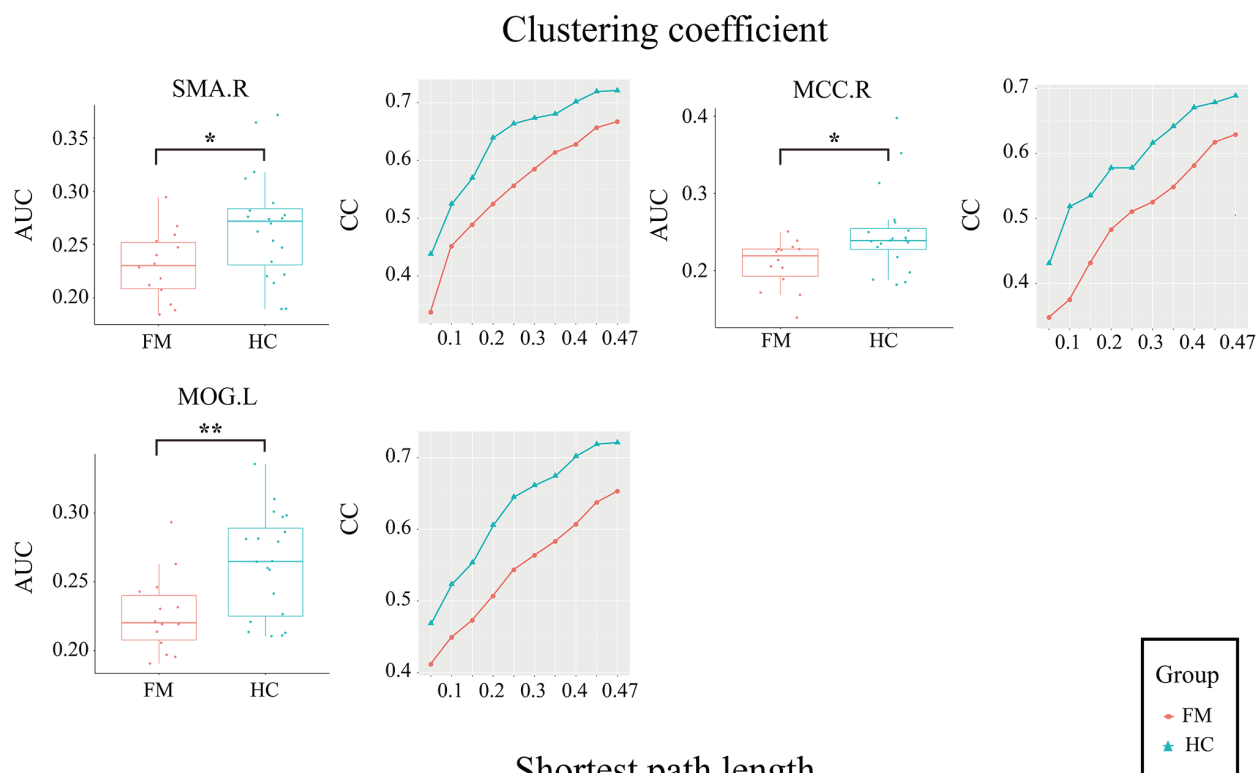

B.

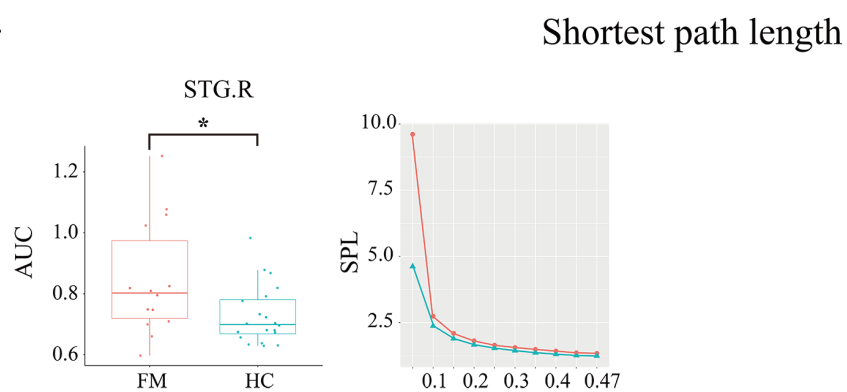

Supplement: S3 Fig — The AUC values of graph theory features were compared, along with the variation trend under the sparse threshold ranging from 0.05 to 0.47. (A) Brain regions with differences in degree centrality; (B) Brain regions with differences in betweenness centrality. AUC, the area under the curve; FM, fibromyalgia; HC, healthy controls; SMA: supplementary motor area; MCC, middle cingulate cortex; MOG, middle occipital gyrus; PCC, posterior cingulate cortex; ANG, Angular gyrus; HES, heschl’s gyrus;; STG, superior temporal gyrus; MTGp, middle temporal gyrus of temporal pole; IFGtri, triangular par of inferior frontal gyrus; SMG, supramarginal gyrus. (PDF) [file pone.0340899.s003.pdf]

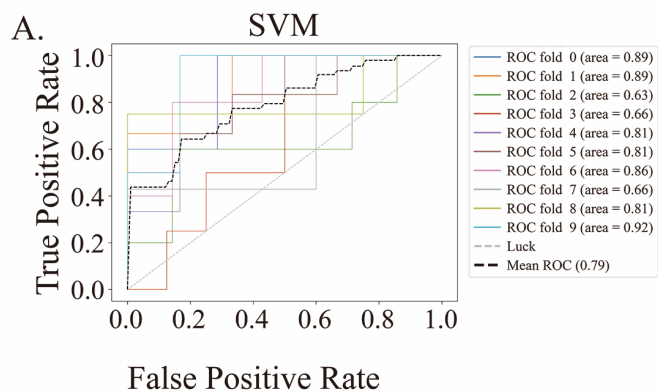

FC

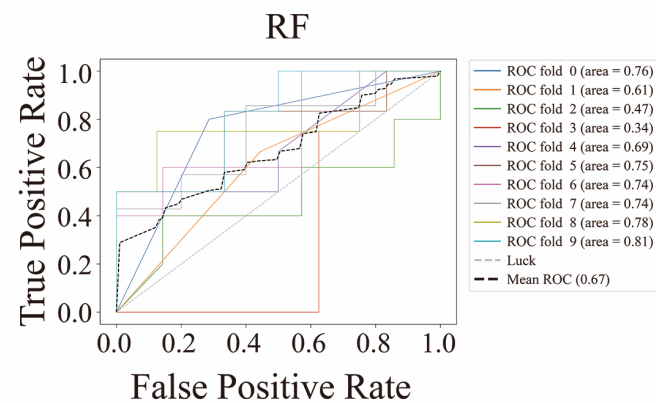

Graph theory + FC

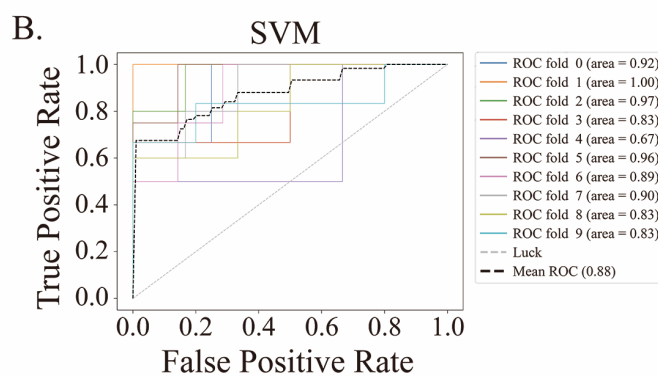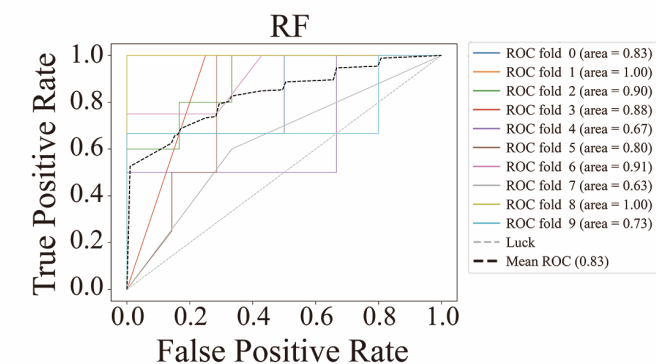

Graph theory of ICA

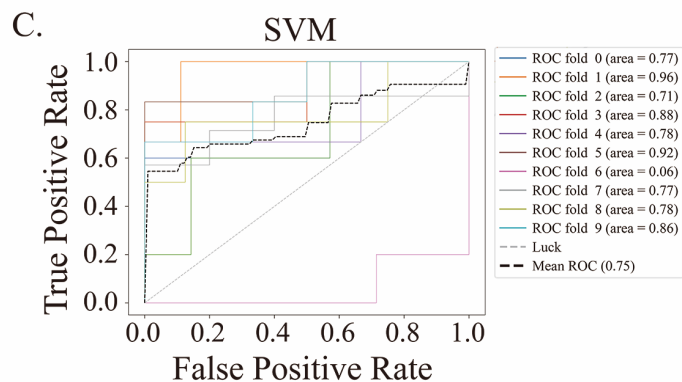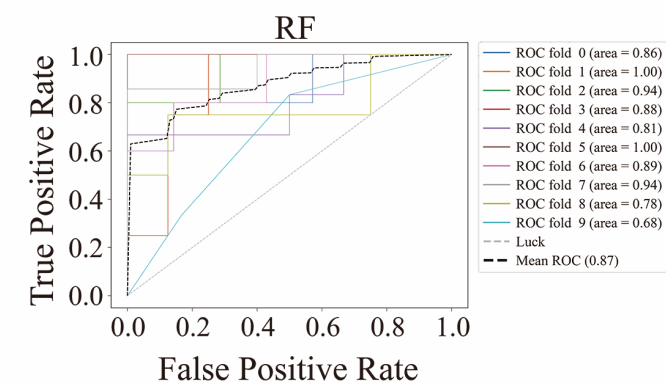

Supplement: S4 Fig — The classification efficiency of imaging features combined with machine learning, as indicated by AUC. (A) Diagnostic classification efficiency of functional connectivity features; (B) Diagnostic classification efficiency of graph theory and functional connectivity features; (C) Diagnostic classification efficiency of ICA. AUC, the area under the curve; ACC, accuracy; FC, functional connectivity; DTI, diffusion tensor imaging; ICA, independent component analysis; SVM, support vector machine; RF, random forest. (PDF) [file pone.0340899.s004.pdf]
