# Supplementary material for: Machine learning for the diagnosis of fibromyalgia based on magnetic resonance imaging
Source: PLoS One. 2026 Feb 2;21(2):e0340899. doi: 10.1371/journal.pone.0340899 (PMC12863509; doi:10.1371/journal.pone.0340899)
Supplement: S1 Table — MFGorb, medial orbital of superior frontal gyrus; CAL, calcarine fissure and surrounding cortex; IFGtri, triangular part of inferior frontal gyrus; CUN, cuneus; LING, lingual gyrus; SOG, superior occipital gyrus; INS, insula; ACC, anterior cingulate and paracingulate gyri; CAU, caudate nucleus; ROL, rolandic operculum; PUT, lenticular nucleus, putamen; PAL, lenticular nucleus, pallidum; SMG, supramarginal gyrus; THA, thalamus; HES, Heschl’s gyrus; STG, superior temporal gyrus; OLF, olfactory cortex; CAU, caudate nucleus. (PDF) [file pone.0340899.s005.pdf]

Brain Region Pairs		t-value
MFGorb.L	CAL.L	3.488
	CAL.R	3.763
	CUN.R	3.543
	LING.L	3.743
	LING.R	3.393
	SOG.L	4.180
	SOG.R	3.389
IFGtri.L	CAL.R	3.245
	CUN.R	3.341
	SOG.L	3.196
IFGorb.L	LING.L	3.518
	SOG.L	3.397
INS.R	ACC.L	3.222
	PUT.L	3.224
	PAL.L	3.175
	THA.L	3.323
	THA.R	3.196
CAU.L	LING.L	3.235
	LING.R	3.394
PUT.L	ROL.R	3.735
	CAL.R	3.199

	HES.L	3.409
PUT.R	ROL.R	3.373
	INS.L	3.375
	HES.L	3.324
PAL.L	ROL.L	3.373
	ROL.R	3.108
	SMG.R	3.326
HES.R	IFGtri.L	3.346
	PUT.L	3.482
	PUT.R	4.054
	PAL.L	3.608
	THA.L	3.914
STG.L	PUT.L	3.650
	PUT.R	3.238
	THA.L	3.267
	THA.R	3.340
STG.R	OLF.R	3.459
	CAU.L	3.468
	PUT.L	4.284
	PUT.R	3.837
	PAL.L	3.727
	THA.L	3.348

	THA.R	3.119
