# Supplementary material for: Machine learning for the diagnosis of fibromyalgia based on magnetic resonance imaging
Source: PLoS One. 2026 Feb 2;21(2):e0340899. doi: 10.1371/journal.pone.0340899 (PMC12863509; doi:10.1371/journal.pone.0340899)
Supplement: S2 Table — SMA: supplementary motor area; MCC, middle cingulate cortex; MOG, middle occipital gyrus; STG, superior temporal gyrus; PCC, posterior cingulate cortex; ANG, Angular gyrus; PCL, paracentral lobule; HES, heschl’s gyrus; MTGp, middle temporal gyrus of temporal pole; IFGtri, triangular par of inferior frontal gyrus; SMG, supramarginal gyrus. (PDF) [file pone.0340899.s006.pdf]

Graph theory characteristic	Brain area	Bonferroni
**Small-world properties**	/	0.035
**Clustering coefficient**	SMA.R	0.021
	MCC.R	0.018
	MOG.L	0.004
**Characteristic path length**	STG.R	0.024
**Nodal Efficiency**	PCC.R	0.004
	ANG.R	0.015
	PCL.R	0.046
	STG.R	0.040
**Nodal Local Efficiency**	SMA.R	0.048
	MCC.L	0.032
	MCC.R	0.017
	MOG.L	0.035
**Degree centrality**	PCC.R	0.028
	ANG.R	0.047
	HES.R	0.038
	STG.R	0.021
	MTGp.R	0.025
**Betweenness centrality**	IFGtri.R	0.019
	SMG.L	0.018
