# Supplementary material for: Machine learning for the diagnosis of fibromyalgia based on magnetic resonance imaging
Source: PLoS One. 2026 Feb 2;21(2):e0340899. doi: 10.1371/journal.pone.0340899 (PMC12863509; doi:10.1371/journal.pone.0340899)
Supplement: S3 Table — DTI, diffusion tensor imaging; SMA, supplementary motor area; CAL, calcarine fissure and surrounding cortex; LING, lingual gyrus; PCL, paracentral lobule; PUT, putamen; PAL, pallidum. (PDF) [file pone.0340899.s007.pdf]

DTI feature	Brain	t-value	BH
**Fractional anisotropy**	SMA.L	4.133	0.006
	SMA.R	4.168	0.006
	CAL.R	3.502	0.019
	LING.R	3.526	0.019
	PCL.L	6.099	< 0.001
	PCL.R	4.292	0.006
**Mean diffusivity**	PCL.L	-3.568	0.031
	PUT.L	-3.326	0.047
	PAL.L	-3.652	0.031
	PAL.R	-4.273	0.012
**Radial diffusivity**	PCL.L	-3.942	0.016
	PUT.L	-3.459	0.047
	PAL.R	-4.087	0.016
