# Supplementary material for: Machine learning for the diagnosis of fibromyalgia based on magnetic resonance imaging
Source: PLoS One. 2026 Feb 2;21(2):e0340899. doi: 10.1371/journal.pone.0340899 (PMC12863509; doi:10.1371/journal.pone.0340899)
Supplement: S4 Table — SMA, supplementary motor area; MCC, middle cingulate cortex; MOG, middle occipital gyrus; PCC, posterior cingulate cortex; ANG, angular gyrus; STG, superior temporal gyrus; IFGtri, triangular par of inferior frontal gyrus; SMG, supramarginal gyrus. (PDF) [file pone.0340899.s008.pdf]

**Graph theory feature types**	**Brain region**
**Clustering coefficient**	SMA.R
	MCC.R
	MOG.R
**Global efficiency**	PCC.R
	ANG.R
**Component efficiency**	SMA.R
	ANG.R
**Degree centrality**	STG.R
**Between centrality**	IFGtri.R
	SMG.R
