# Supplementary material for: Machine learning for the diagnosis of fibromyalgia based on magnetic resonance imaging
Source: PLoS One. 2026 Feb 2;21(2):e0340899. doi: 10.1371/journal.pone.0340899 (PMC12863509; doi:10.1371/journal.pone.0340899)
Supplement: S5 Table — DTI, diffusion tensor imaging; MFG, middle frontal gyrus; FG, fusiform gyrus; PAL, pallidum; STG, superior temporal gyrus; MTGp, middle temporal gyrus of temporal pole; ITG, inferior temporal gyrus. (PDF) [file pone.0340899.s009.pdf]

**DTI feature**	**Brain region**
**Fractional anisotropy**	MFG.R
	FG.R
	PAL.L
	STG.R
	MTGp.R
**Radial diffusivity**	MFG.R
	ITG.L
**Mean diffusivity**	MFG.R
	STG.R
	ITG.L
